# Effects of Platelet-Rich Fibrin on Hard Tissue Healing: A Histomorphometric Crossover Trial in Sheep

**DOI:** 10.3390/ma13071695

**Published:** 2020-04-04

**Authors:** Esra Ondur, Nilufer Bolukbasi Balcioglu, Merva Soluk Tekkesin, Ozlem Guzel, Selim Ersanli

**Affiliations:** 1 Department of Oral Implantology, Faculty of Dentistry, Istanbul University, Istanbul 34093, Turkey; dt.esrayildirim@gmail.com (E.O.); selimersanli@gmail.com (S.E.); 2 Department of Tumor Pathology, Institute of Oncology, Istanbul University, Istanbul 34093, Turkey; msoluk@istanbul.edu.tr; 3 Department of Surgery, Veterinary Faculty, Istanbul University Cerrahpasa, Istanbul 34320, Turkey; drozlemguzel@gmail.com

**Keywords:** platelet rich fibrin, anorganic bovine bone, bone augmentation, histological analysis

## Abstract

Bone defects lead to aesthetic and functional losses, causing dental rehabilitation to be more difficult. The objective of this work is to histologically assess the hard tissue healing of bone defects filled with platelet-rich fibrin (PRF) alone or as an adjuvant for mixing with and covering anorganic bovine bone (ABB), compared to ABB covered with a resorbable collagen membrane (CM). This study was designed as a crossover animal study. Four 5-mm tibia defects, 5 mm apart from each other, were surgically created on the tibias of 6 sheep. The defects were randomly filled with ABB + CM; PRF alone; ABB+PRF; or were left empty. The animals were euthanized on days 10, 20, and 40 post-operatively. No group showed any signs of bone necrosis. Inflammation was observed in 2 control and 3 test defects with no statistically significant difference between groups at each time point. The ABB + CM and ABB + PRF groups experienced the highest bone regeneration ratios. No differences between the empty-defect and PRF groups were observed in regard to bone regeneration. No statistical difference was observed between the ABB+PRF and ABB + CM groups in regard to bone regeneration and the amount of residual graft material at each time point. The use of PRF should be preferred due to its autogenous origin, low cost, and ease of use.

## 1. Introduction

A fast resorption process occurs following the loss of natural teeth due to trauma, periodontal disease, caries or extractions [1,2,3]. The severity of resorption increases as the duration of edentulism extends. Placement of dental implants in ideal positions becomes harder or even impossible owing to both the vertical and horizontal resorptions. Guided bone regeneration techniques and graft materials of various features (autogeneous graft, allograft, xenografts and alloplasts) and membranes (resorbable and non-resorbable) are used to provide bone regeneration. Today, to reduce the time for new bone formation and to increase the quantity and quality of the newly formed bone growth factors are usually combined with these augmentation materials [1,2,3].

Platelet-rich fibrin (PRF) is a second-generation platelet concentrate, and has been used in various dental surgical procedures since 2000 [4]. The major advantages of PRF are that it is a completely autogenous, easy-to-prepare biomaterial and that it releases high amounts of growth factors for a relatively long period [4,5,6,7]. The fibrin-rich matrix obtained through this method was recently classified as L-PRF due to its leukocytes content [8]. It was observed that the fibrin matrix enhances PRF’s healing capacity by supporting leukocytes and cytokines [9]. Also, the fibrin matrix effectively triggers angiogenesis and the tissue-healing processes [10]. Neutrophils within the fibrin clot phagocytose foreign particles and bacteria in wounds [11]. The PRF also contains macrophages that are effective in the healing process. Moreover, Kobayashi et al. [7] showed that a transforming growth factor (TGF-β1), vascular endothelial growth factor (VEGF), and platelet-derived growth factor (PDGF-AB) are continuously released within L-PRF for seven days. Due to the accelerating effects of these cells, cytokines, and growth factors on the healing process in soft and hard tissues, L-PRF may be used in areas such as socket-preservation techniques, periodontal surgery, sinus-lifting surgery, and bone reconstruction procedures in dentistry.

The PRF has been used alone as a graft material or a membrane, or mixed with other graft materials in various bone augmentation procedures [12,13]. ABB today still represents one of the most popular grafting materials due to its human-bone-like structure, slow resorption time that leads to maintained volume during healing, induction of bone regeneration, and status as a well-documented biomaterial [13,14,15,16,17]. In many clinical and experimental studies, ABB and PRF were used together for various augmentation procedures in oral surgery [12,18,19,20,21,22,23,24].

This study aims to histologically evaluate the hard tissue healing of bone defects filled with platelet-rich fibrin (PRF) alone or as an adjuvant for mixing and covering anorganic bovine bone (ABB), compared to ABB covered with a resorbable collagen membrane (CM), in the early and late periods. The healing process of the bone defects created in sheep was histologically and histomorphometrically observed post-operatively at days 10, 20, and 40 in a crossover trial. A clinical study was conducted to test the following null hypothesis: using PRF alone or with different grafting materials and resorbable membranes in the treatment of bone defects do not yield a statistically significant difference in terms of inflammation, new bone area and residual graft area.

## 2. Materials and Methods

### 2.1. Study Design

This animal study was designed as randomized crossover trials, since every individual received both test and control procedures. The research protocol was approved by the Animal Experimentation Ethics Committee of the İstanbul University (2011/92). All the procedures were performed in accordance with the İstanbul University’s Ethical Guidelines for the Care and Use of Laboratory Animals.

**Figure 1:**
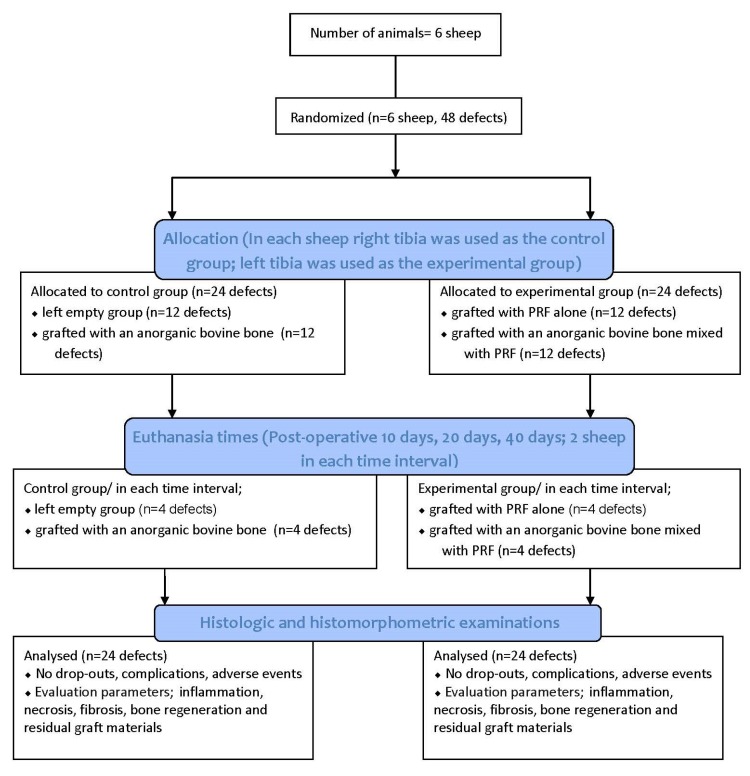
Flow diagram of the study. PRF: Platelet Rich Fibrin.

This manuscript was prepared according to the guidelines proposed by the ARRIVE [25] (Figure 1).

### 2.2. PRF Preparation

Following the general anesthetic administration, the venous blood was drawn from the jugular vein of each animal into 10-mL tubes (Vacuette, Grenier Bio-One, Kremsmünster, Austria), 8 tubes in total. The tubes were then centrifuged for 12 min at 2700 rpm (~710 × *g*, PC-02, Process, Nice, France), according to the protocol suggested by Choukroun and co-workers [10]. Following the centrifuge procedure, a PRF clot accumulated between the supernatant acellular plasma and erythrocyte at the bottom. The fibrin clot and red corpuscles were removed from the tube with a scalpel. ed corpuscles were separated from the fibrin clot by the use of scissors. The fibrin clot obtained from the supernatant and erythrocyte through use of a sterile tweezer was cut into small pieces with scissors and mixed with graft in a ratio of 0:50. The remaining fibrin was turned into a solid covering membrane with the help of a metal box.

### 2.3. Experimental Animals

Six male sheep, taken from Istanbul University Cerrahpasa, Faculty of Veterinary Medicine were included in this study (mean weight: 42.3 ± 1.86 kg; mean age: 2.5 ± 0.54 years). The surgical procedures were performed under sterile conditions and general anesthesia at Istanbul University Cerrahpasa, Faculty of Veterinary Medicine, Department of Surgery.

The animals were fasted 24 h prior to surgical operation. One hour prior to the surgical procedures, all the animals were intramuscularly injected with Ceftriaxone sodium (2 mg/kg; 1 g, Iesef, Ulagay, Istanbul, Turkey) and diclofenac potassium (Dikloran, Deva İlaç, Istanbul, Turkey). At every surgical session the animals were anesthetized. Preanesthetic xylazine (0.2–0.5 mg/kg IM, Rompun, Bayer, Istanbul, Turkey) was administered to establish vascular access. The animals were then intubated following intravenous administration of ketamine hydrochloride (5 mg/kg, Ketalar, Eczacıbaşı, Istanbul, Turkey) as a preanesthetic. General anesthesia was achieved with 3.5% isoflurane (Forane, Abbott) and sustained with 1.5% isoflurane. Both tibias were sheared and then cleansed with 10% povidione-iodine.

After surgeries, the animals were placed in separate cages and fed a standard diet. During the study period, the sheep were examined for leg fractures and their general health status. The animals were randomly euthanized on day 10, 20, and 40 (two animals at each time interval) using an overdose of sodium pentothal (30 mg/kg, IV, Abbott, Istanbul, Turkey). The tibias were carefully dissected free from soft tissues and hard-tissue samples were transferred immediately into 10% buffered formalin.

### 2.4. Surgical Procedures

**Figure 2:**
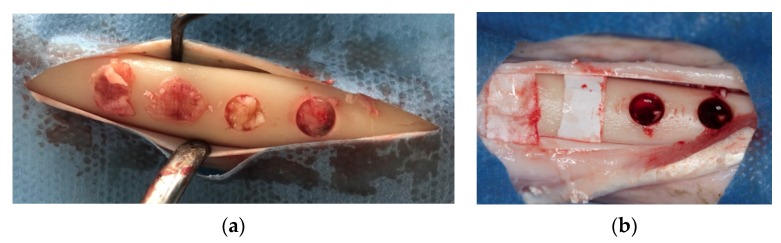
Defects in test and control groups. (**a**): test group; (**b**): control group.

Skin and periosteal incisions were performed on left and right tibia to achieve surgical field exposure. Four 5-mm diameter defects, 5-mm apart from each other, were created using a calibrated 5 × 22 mm trephine bur (Salvin Dental Specialties, Inc., Charlotte, NC, USA) to a depth of 5 mm, using a physiodispenser at 1500 rpm and under cold saline irrigation. The whole superficial cortical plate was removed. Randomly, two of the defects created on the right tibia (control) were left empty, and the other two were grafted with an anorganic bovine bone (Bio-Oss, Geistlich PhaRMA, Wolhusan, Switzerland) and then covered with a resorbable collagen membrane (Bio-Gide, Geistlich PhaRMA). On the left tibia (test), two defects were filled with PRF alone whereas the other two were filled with anorganic bovine bone (Bio-Oss, Geistlich PhaRMA, Wolhusan, Switzerland) mixed with PRF, and covered with a PRF membrane (Figure 2a,b). The subcutaneous tissue was closed with 2.0 resorbable sutures (Vicryl, Ethicon Inc., New Jersey, USA) and the skin was closed with skin stapler.

### 2.5. Histologic and Histomorphometric Outcomes

Histologic and histomorphometric examinations were performed by a single blinded examiner (M.S.T.). The received specimens were fixed in 10% buffered formalin for a period of 1 week. Following the fixation process, the specimens were decalcified by a solution of 50% formic acid and 20% sodium citrate. The decalcified specimens were then embedded into paraffin and cut into 5- to 7-micron sections, following a routine tissue processing. The sections were stained with hematoxylin-eosin and examined under a light microscope (Olympus BX60, Tokyo, Japan). The sections were examined at 10× and 20× magnification (BX60 microscope, Olympus Corporation, Tokyo, Japan) to evaluate inflammation, necrosis, and fibrosis. Histomorphometric evaluations of bone regeneration and residual graft materials were also performed. Images of 4 chosen areas of each defect were taken at 10× under the same microscope and then computerized. These operations were performed by a camera (E-330 camera, Olympus Corporation, Tokyo, Japan) connected to the microscope and a computer. The images were processed by the Olympus Soft Imaging System Analysis Five software (Olympus Corporation, Tokyo, Japan) on the computer to measure the area occupied by bone regeneration and residual graft, and the measurements were compared with the 2.27-mm^2^ total area.

### 2.6. Randomization Procedure

Sample size was not calculated. It was decided to prepare 48 experimental sites on 6 sheep (eight sites for the sheep; four sites for each procedure and time points). The sheep randomly received the test and control procedures. Nevertheless, the right tibias were used as control sites and the left tibias were used as experimental sites for all of the animals. A blinded statistician generated the allocation sequence and assigned procedures to sites. Assignment was performed using opaque envelopes containing the generated unique randomization code opened immediately after the defects were created. Two pre-generated random lists, consisting of a randomized sequence of consecutive numbers matching the different procedures within test and control group, and the sequence for the euthanized sheep, were created using Random number generator pro 1.91 for Windows (Segobit Software, Redmond, Washington, USA). Opaque envelopes containing the randomization codes were sequentially numbered and sealed. In accordance with a pre-generated list, an independent clinician, not previously involved in the trial, prepared all the envelopes. Data were collected on spreadsheets (Excel software, Microsoft Corporation, Redmond, Washington, USA).

### 2.7. Statistical Analysis

The methodology was reviewed by an independent statistician not previously involved in the study. The NCSS (Number Cruncher Statistical Systems) 2007 (Kaysville, Utah, USA) software was used for the statistical analysis. The Mann-Whitney *U* test was used to assess quantitative data for the presence of a difference between two groups for non-normally distributed parameters along with the descriptive statistics methods (mean, standard deviation, median, frequency, ratio, minimum, and maximum) to assess the research data. The Kruskall Wallis test was used for the comparison of three or more non-normally distributed groups, and the Bonferroni-adjusted Mann-Whitney *U* test was used for the pairwise comparison. Chi-square test was used for comparison of quantitative data. Differences were considered significant at a *p* value <0.05. 

## 3. Results

### 3.1. Clinical Findings

In total, 48 defects in 6 sheep were collected and histologically examined. All the animals were healthy before being euthanized. No dropouts were experienced, and no complications or adverse events were observed at any stage of the research.

### 3.2. Histological Findings

**Figure 3:**
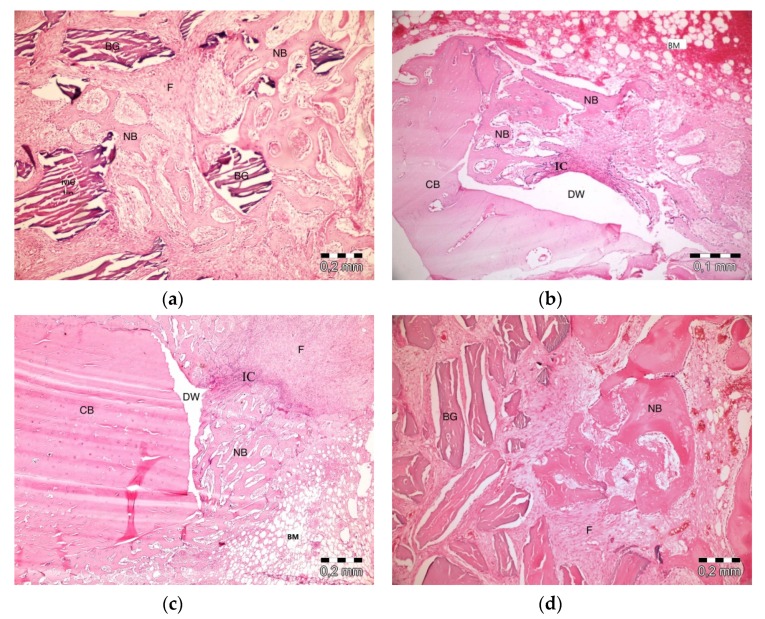
Histologic findings on the 10th day. (**a**): platelet-rich fibrin (PRF) + anorganic bovine bone (ABB); (**b**): PRF; (**c**): Empty Defect; (**d**): ABB + collagen membrane (CM). N: New bone area; F: Fibrosis; BG: Bone graft; DW: Defect wall; CB: Compact bone; BM: Bone marrow; IC: inflammatory cells infiltration.

After 10 days, the graft particles were observed to be sustained in defects in which PRF and ABB were used in combination. Areas of bone regeneration were present between graft particles. In some areas, fibrosis was observed, especially around the graft material. No signs of necrosis or inflammation were observed (Figure 3a). Bone regeneration in the PRF group was observed on the side sections of the defect areas. Sporadic bleeding was observed. No signs of necrosis were observed. A defect showed lymphocytic infiltration with moderate initial inflammation. Loose connective tissue was observed in the defect areas resembling a control group (Figure 3b). Bone regeneration in the empty group was observed only around the defect areas. Moderate inflammation in a defect in the control group was observed, especially in its upper parts. No signs of necrosis were observed. Fibrosis was partly observed, especially in the center section of a defect, but mainly, loose, venous connective tissue was observed (Figure 3c). Bone regeneration was barely seen around the graft particles in the ABB+CM group. Inflammatory cell infiltration and slight fibrosis with partly venous-rich areas were observed in one defect. Necrosis was not present (Figure 3d).

**Figure 4:**
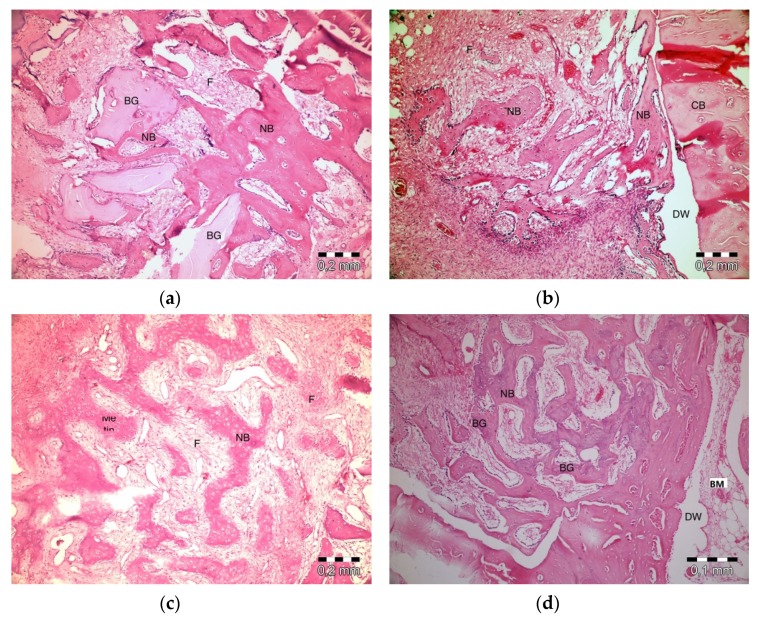
Histologic findings on the 20th day. (**a**): PRF + ABB; (**b**): PRF; (**c**): Empty Defect; (**d**): ABB + CM. N: New bone area; F: Fibrosis; BG: Bone graft; DW: Defect wall; CB: Compact bone; BM: Bone marrow.

After 20 days, slight inflammation was observed in the PRF + ABB group. No signs of necrosis were observed. Fibrosis was observed in bone regeneration areas and around the residual graft material. Graft particles were observed to shrink and be surrounded by bone (Figure 4a). Inflammation or necrosis was not observed in the PRF group. Fibrosis was evident. Bone regeneration areas were observed to expand from the walls of the defect to its center. A thinner trabecular bone structure was observed in the central section (Figure 4b). No inflammation or necrosis was observed in the empty-defect group. A fibrovascular structure was notable in the central section of a defect. Increased bone regeneration activity and, in part, bone marrow formation was observed (Figure 4c). Fibrosis was evident in the central part of defects in ABB + CM group. Inflammation or necrosis was not observed. The thinning of graft particles was observed, and the surrounding new bone trabeculae became more evident (Figure 4d).

**Figure 5:**
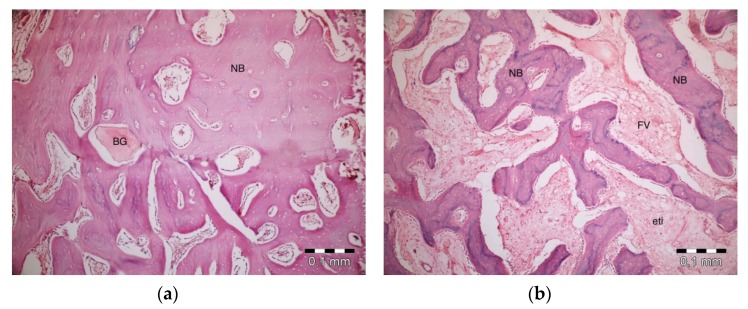

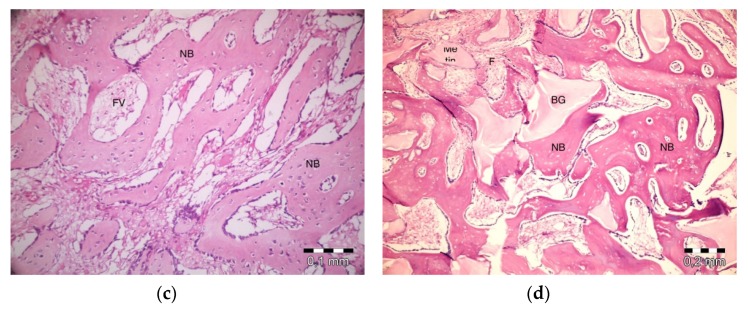
Histologic findings on 40^th^ day. (**a**): PRF + ABB; (**b**): PRF; (**c**): Empty Defect; (**d**): ABB + CM. N: New bone area; F: Fibrosis; BG: Bone graft; DW: Defect wall; CB: Compact bone; BM: Bone marrow; FV: Fibrovascular area.

After 40 days, necrosis was not observed in the PRF + ABB group. Inflammation was present in one defect. Fibrosis was almost completely replaced by bone regeneration and partly by bone marrow. Graft particles became much thinner, and the surrounding bone trabeculae became thicker. Thicker areas became compact bone. Some vascular structures were observed (Figure 5a). No inflammation or necrosis was present in the PRF defects. However, the progression of fibrosis was partly observed. Lamellar bone formation was observed to fill a large part of the defect area (Figure 5b). No inflammation or necrosis was observed in the defects in the empty-defect control group. Fibrosis seemed to decrease. An increase in lamellar bone formation was observed, but the defect was not completely filled with bone (Figure 5c). No signs of inflammation or necrosis were observed in the ABB + CM group. Graft particles that were smaller in size compared to those observed on day 20 were observed in the defect area. Bone regeneration areas with partial fibrovascular tissue became thicker around the graft material (Figure 5d).

### 3.3. Histomorphometric Findings

No statistically significant difference was observed between groups on day 10, 20, and 40 in regard to inflammation (*p* > 0.05). On day 10, one defect in each of the empty defect, PRF and PRF + ABB groups showed inflammation. On days 20 and 40, only one defect in PRF + ABB group showed inflammation. 

Table 1 and Table 2 show the bone regeneration ratio in 4 groups at 3 time intervals. On day 10, the lowest bone regeneration ratio was observed in the empty-defect group whereas the highest bone regeneration ratio was observed in the PRF + ABB group. The groups did not have a statistically significant difference in regard to the bone regeneration measurements on day 10 (*p* = 0.0061). According to a pairwise comparison that determined the different group bone regeneration measurements, the bone regeneration measurement of the PRF+ABB group was significantly higher than that of the empty-defect group (*p* = 0.091). No significant difference was found between the ABB + CM group and the empty-defect group in regard to bone regeneration measurements (*p* = 0.0755); it was noteworthy that the measurements for the ABB + CM group were higher. No statistically significant difference was observed in other pairwise comparisons (*p* > 0.05). On day 20, a statistically significant difference between groups was observed in regard to day 20’s bone regeneration measurements (*p* = 0.0115). Statistically insignificant bone regeneration ratios (47.00 ± 6.27 versus 45.75 ± 3.77) were observed in the empty-defect and PRF groups. According to pairwise comparisons, the bone regeneration ratio of the PRF + ABB group was found to be significantly higher than that of the PRF group (*p* = 0.0455). High bone regeneration activity in the PRF + ABB group was observed in comparison to that of the empty-defect group, but it was not statistically significant (*p* = 0.0765). No statistically significant difference was observed in other pairwise comparisons (*p* > 0.05). On day 40, the highest bone ratios were measured in the PRF + ABB (80.50 ± 2.08) and ABB+CM (82.50 ± 6.61) groups, and the difference is not statistically significant. A statistically significant difference was observed in the comparison of 4 research groups (*p* = 0.0051). According to the pairwise comparison used to determine the ratios for different groups, the bone regeneration ratio for the empty-defect group was found to be significantly lower (*p* < 0.05) than the ratios of the PRF+ABB (*p* = 0.023) and ABB + CM (*p* = 0.014) groups. No statistically significant difference was observed in other pairwise comparisons (*p* > 0.05). The bone regeneration ratio in the empty-defect and PRF groups on day 40 were 53.50 ± 3.70 and 67.25 ± 3.86, respectively.

Table 3 shows the ratios for the residual graft materials in the ABB+CM and PRF+ABB groups. No significant difference was observed between these two groups at each of the 3 time intervals (*p* > 0.05).

## 4. Discussion

Over the past decades dental implants have become a very popular treatment for partial or total edentulism. The most important prerequisite for implant placement is sufficient bone volume. Several factors such as periodontal disease, extractions, congenital abnormalities or trauma may cause jaw bone deficiencies [1,2,3]. Natural or synthetic grafting materials and resorbable or non-resorbable membranes are routinely used to gain enough bone volume to ensure osseointegration. Currently, there is not enough data on which material is superior. Autologous grafts are considered the ‘gold standard’ due to being biocompatible and autogenous. In cases where autogenous grafts are not preferred, allografts and xenografts are frequently used. In intraosseous defects, the effects of the use of PRF along with ABB on hard-tissue healing in the post-operative early and late periods were evaluated histologically and histomorphometrically in this study. The highest bone regeneration ratio was observed in the ABB + CM and PRF + ABB groups. No statistically significant difference was observed between these groups in regard to bone regeneration and the amount of the residual graft material at any of the three time intervals. 

To the best of our knowledge, at the time of writing this article, there are few experimental studies comparing PRF alone or PRF combined with ABB. This makes it difficult to evaluate whether the present results are consistent with other comparable studies. Mazor et al. [26] and Tajima et al. [27] reported that the use of PRF alone as a graft material in sinus augmentation resulted in the achievement of sufficient naturally regenerated bone in the sinus cavity and that the embedded implants were stable. In this study, the comparison of the defects augmented with PRF alone and the empty defects revealed that similar results were observed in both groups on day 20, whereas statistically insignificantly higher bone regeneration was observed on day 10 and day 40 in the PRF group. However, the bone regeneration percentages in the empty-defect and PRF groups were lower than that of the ABB group. An evaluation of the effect of bone regeneration when different graft materials are used with PRF on the osseointegration of dental implants will be helpful in future experimental researches.

A critical-sized defect of 5 mm in diameter was surgically created on 48 rats by Oliveira et al. [19]. Homogenous clot, autogenous clot, autogenous PRF, homogenous PRF, ABB or PRF along with ABB were applied to the defects. Histomorphometric analyses on day 30 and 60 showed that bone regeneration percentage was significantly higher in the PRF + ABB (54.05%) group than in all of the other groups (54.05% and 57.34%, respectively). The study revealed that PRF increases bone regeneration only in combination with anorganic bovine bone. Bolukbasi et al. [18] compared the success of PRF + ABB and ABB + CM in 32 two-stage sinus surgeries on 25 patients. Histologic and histomorphometric evaluations were performed on the samples collected from prepared implant cavities after 6 months. No statistical difference was observed in both groups in regard to bone regeneration and residual graft material. Yoon et al. [28] created 2 defects in the calvariae of rabbits and filled 1 defect with ABB and the other with ABB + PRF. The animals were euthanized on week 1, 2, and 4; bone regeneration was evaluated through a histomorphometric analysis, and the expression of the VEGF (Vascular Endothelial Growth Factor) was determined by immunohistochemical staining. No difference was observed for both parameters between the experimental and control groups. The researchers noted that PRF use along with xenograft made no significant difference in regard to bone regeneration. In a similar study, Choukroun et al. [29] augmented 6 sinuses with a PRF + allograft combination and 3 sinuses with allograft alone. Histological samples were collected in month 4 from the PRF applied group and in month 8 from the control group. Similarities between the histological maturation in the experimental group in month 4 and in the control group in month 8 as well as similar amounts of bone regeneration in both groups were observed. 

Possible reasons for the different results in the clinical and experimental studies in which PRF and various graft materials were used in combination might be as follows: different observation periods, the types and characteristics of the used graft materials, the centrifuge used in the process, and the types of experimental animal. In the present study, an osseoconductive xenograft material was used. This grafting material is also known to be biocompatible due to its preparation technique. During its preparation the protein and lipid components are eliminated and therefore it does not run intolerance or infection risks. Being a totally autogeneous and biocompatible biomaterial and due to the growth factors, fibrins, and cytokines it contains, applying PRF along with xenograft might give the graft material an osseoinductive characteristic [30,31]. Conducted studies showed that PRF releases growth factors such as TGF-β1, PDGF-β, and VEGF, particularly in the first 7 days [6,32] and continues to release them in decreasing amounts for up to 28 days [33]. The relatively long process of collecting histologic and histomorphometric samples in clinical studies due to ethical reasons makes it impossible to evaluate PRF’s effect on the early stage of the healing process. The result of the present study might be affected by the type and number of the experimental animal used in the experimental studies conducted to evaluate the early stage and different intervals of the healing process. The time it takes to collect enough amounts of venous blood from experimental animals, such as rabbits and rats, is relatively long and their blood structure differs from that of humans. Also, the venous blood should be collected in empty tubes with no anticoagulants and should rapidly be centrifuged in order to obtain PRF successfully, which is hard to achieve with low-weight experimental animals [34]. In this study, sheep were preferred as experimental animals because their blood resembles human blood. It was possible to collect enough venous blood for the centrifuge and prepare defects of a standard diameter in the same regions (5-mm diameter in the tibias), According to a recent classification, the PRF produced with this technique was named L-PRF due to the leukocytes it contains [8]. Today, different devices are used to produce PRF. However, the characteristics of the centrifuge and centrifugation protocol change the attributions and fibrin structures of the PRF produced, so PRFs produced with different techniques or devices are expected to have different levels of efficacy [33,35].

Membranes are used to prevent the invagination of the mucogingival tissues and stabilize the graft material in augmented areas. Resorbable collagen membranes are biocompatible, easy-to-apply, and clinically proven biomaterials. In cases in which collagen membranes are used, issues with the primary closure of the edge of the wound or perforation of the membrane might be observed in the early period. PRF is reported to be useable as a membrane in augmented areas such as socket preservation, sinus augmentation and guided bone regeneration due to its status as an autologous fibrin matrix with high cytokine and growth factor contents [10,18,36]. In this study, the surface of the defect area was covered with PRF membranes in the PRF + ABB group and with CM in the ABB + CM group. No perforation in the soft tissue was observed in either group. Also, a higher bone regeneration ratio was observed in all measurement intervals in PRF + ABB and ABB + CM groups in comparison to the PRF and ABB groups. Gassling et al. [37], covered the lateral window with PRF or CM membranes in 2-stage bilateral sinus-floor augmentation surgeries in 6 patients. Similar mean values of vital bone formation and residual bone substitute were observed in both groups in the histomorphometric analysis at the end of the fifth month. No dehiscence and membrane exposures were observed in either group. Bolukbasi et al. [18] covered the lateral window with PRF or CM in a 2-stage bilateral sinus-floor augmentation surgery. Similar to Gassling et al. [37], no difference between the two groups in new bone formation and biomaterial remnant was observed.

There are some limitations of the present study: First; the number of experimental animals was determined in accordance with the recommendation of the ethics committee in such a way that the least experimental animals were used and the sample size not calculated. Second, biochemical composition of sheep blood is not exactly the same as humans. The amount and the content of PRF produced from the venous blood of sheep might differ from human depending on the amount of the thrombocytes in the blood. Also, the growth factors and the amount of the cytokines contained within the PRF were ignored in this study. The evaluation of the amount of the thrombocytes in the blood and the content of the growth factors and cytokines contained within the produced PRF will allow a more precise evaluation of the results in future studies. Third, four 5-mm diameter defects, 5-mm apart from each other, were created in each tibia. It is useful to evaluate the effectiveness of the PRF+ABB combination in critical size diameter defects. Fourth, this study aimed to evaluate the hard tissue healing of bone defects filled with PRF, PRF + ABB or ABB + CM. The effectiveness of the combination of different grafting materials and PRF could produce distinct features.

## 5. Conclusions

The results of this experimental animal study showed that mixing and covering the ABB with PRF provides a similar bone regeneration pattern compared to the use of ABB in combination with a collagen membrane. The use of PRF may be preferred due to its autogenous origin, being cheaper than collagen membrane, and ease of use. Further randomized controlled trials in human are needed to confirm these preliminary results. In order to evaluate the effectiveness of PRF especially in the early period, it would be beneficial to use PRF in clinical studies to allow histological samples to be taken in earlier periods, such as socket prevention technique.

## Figures and Tables

**Table 1:** Bone regeneration ratios in each group on day 10, 20, and 40.

New Bone Area	Empty Defect (n = 4)	PRF (n = 4)	PRF + ABB (n = 4)	ABB + CM (n = 4)	^a^P
	Min–Max (Median) Mean ± Std	Min–Maxs (Median) Mean ± Std	Min–Maxs (Median) Mean ± Std	Min–Maxs (Median) Mean ± Std	
Day 10^th^ (%)	22–32 (27)	27–41 (37.5)	50–58 (55.5)	44–55 (52.5)	0.006*
	27.00 ± 4.16	35.75 ± 6.40	54.75 ± 3.40	51.00 ± 5.23	
Day 20^th^ (%)	40–55 (46.5)	41–50 (46)	65–68 (66.5)	55–69 (63)	0.011*
	47.00 ± 6.27	45.75 ± 3.77	66.50 ± 1.29	62.50 ± 6.24	
Day 40^th^ (%)	50–58 (53)	63–71 (67.5)	78–83 (80.5)	75–89 (83)	0.005*
	53.50 ± 3.70	67.25 ± 3.86	80.50 ± 2.08	82.50 ± 6.61	
^a^p	0.014*	0.008*	0.007*	0.009*	
^b^P ^Day 10–Day 20^	0.208	0.422	0.350	0.500	
^b^P ^Day 10–Day 40^	0.011*	0.006*	0.005*	0.007*	
^b^P ^Day 20–Day 40^	0.840	0.315	0.350	0.280	

^a^ Kruskall Wallis Test; ^b^ Bonferroni-adjusted Mann Whitney U Test; * *p* < 0.05.

**Table 2:** Pairwise comparison of groups.

Groups		Day 10	Day 20	Day 40
		^b^p	^b^p	^b^p
Empty Defect	PRF	1.000	1.000	1.000
	PRF + ABB	0.009*	0.076	0.023*
	ABB + CM	0.075	0.223	0.014*
PRF	PRF + ABB	0.138	0.045*	0.526
	ABB + CM	0.654	0.169	0.380
PRF + ABB	ABB + CM	1.000	1.000	1.000

^b^ Bonferroni-adjusted Mann Whitney U Test; * *p* < 0.05.

**Table 3:** Residual graft ratios in both groups on the 10th, 20th, and 40th days.

Residual Graft (%)	PRF + ABB (n = 4)	ABB + CM (n = 4)	^a^P
	Min–Max (Median) Mean ± Std	Min–Max (Median) Mean ± Std	
Day 10^th^ (%)	25–30 (27.5)	29–35 (31)	0.057
	27.50 ± 2.08	31.50 ± 2.65	
Day 20^th^ (%)	19–21 (20.5)	20–31 (25.5)	0.200
	20.25 ± 0.96	25.50 ± 5.80	
Day 40^th^ (%)	14–16 (15)	3–20 (12)	0.686
	15.00 ± 0.82	11.75 ± 7.37	
^a^p	0.007*	0.020*	
^b^p ^Day 10-Day 20^	0.346	0.206	
^b^p ^Day 10-Day 40^	0.005*	0.018*	
^b^p ^Day20-Day 40^	0.346	1.000	

^a^ Kruskall Wallis Test; ^b^ Bonferroni-adjusted Mann Whitney U Test; * *p* < 0.05.
